# Spread of an Experimental *Salmonella* Derby Infection in Antibiotic-Treated or *Lawsonia intracellularis* Vaccinated Piglets

**DOI:** 10.3390/ani8110206

**Published:** 2018-11-12

**Authors:** Christian Visscher, Jasmin Mischok, Saara Sander, Jutta Verspohl, Eva-Ursula Peitzmeier, Isabel von dem Busche, Josef Kamphues

**Affiliations:** 1 Institute for Animal Nutrition, University of Veterinary Medicine Hannover, Foundation, Bischofsholer Damm 15, D-30173 Hanover, Germany; jasminmischok@gmx.de (J.M.); saara@sander-s.de (S.S.); josef.kamphues@tiho-hannover.de (J.K.); 2 Institute for Microbiology, University of Veterinary Medicine Hannover, Foundation, Bischofsholer Damm 15, D-30173 Hannover, Germany; jutta.verspohl@tiho-hannover.de; 3 Tierarztpraxis Dr. Peitzmeier, Meente 24, D-32479 Hille, Germany; info@tierarztpraxis-peitzmeier.de (E.-U.P.); vondembusche@tierarztpraxis-peitzmeier.de (I.v.d.B.)

**Keywords:** antibiotics, caecum, infection, lymph nodes, *Lawsonia intracellularis*, *Salmonella* Derby, vaccination

## Abstract

**Simple Summary:**

Today, pigs repeatedly suffer from diarrhoea which requires treatment with antibiotics. Infections with *Lawsonia intracellularis* are one of the most common diseases, which, although usually subclinical, have a negative impact on performance. The alternative to antibiotic treatment is vaccinations against *Lawsonia intracellularis*, not least because antibiotic treatments are suspected of promoting the spread of certain zoonotic pathogens. A study was carried out with piglets from a farm that had a problem with *Lawsonia intracellularis* infections. In half of the animals, antibiotic treatments with tylosin were carried out in piglet rearing. In the other group, the piglets had been vaccinated against *Lawsonia intracellularis* as suckling piglets. Individual animals from both groups were subsequently artificially infected with *Salmonella* as piglets. A total of 72 animals were included in the study, 12 of which were primarily infected. The other animals had the possibility of becoming infected via direct animal contact or the faeces of infected animals. The detection of *Salmonella* in stool and intestinal lymph nodes was significantly higher in animals previously treated with antibiotics. Treatment with tylosin may significantly increase the spread of the *Salmonella* infection not observed after early *Lawsonia intracellularis* vaccination.

**Abstract:**

*Lawsonia intracellularis* infections are a common reason for antibiotic treatment in pig production. Experimental studies in animals naturally infected with *Lawsonia intracellularis* comparing the course of an experimental *Salmonella* infection in piglets previously treated with tylosin or vaccinated against *Lawsonia intracellularis* are scarce. A total of 72 seven-week-old *Salmonella*-free pigs were taken from a herd with a *Lawsonia intracellularis* history in piglet rearing. The pigs were divided into two groups with three replicates each. Animals had either been previously treated with tylosin (10 mg/kg body weight) for seven days (AB^+^VAC^−^) or had been vaccinated as suckling pigs by drenching (Enterisol^®^Ileitis; AB^−^VAC^+^). Two animals per replicate were primarily infected with *Salmonella* Derby (1.04 × 10^8^ colony-forming units per animal). The detection of *Salmonella* in faeces (*p* < 0.0001, odds ratio: 3.8364) and in the ileocaecal lymph nodes (*p* = 0.0295, odds ratio: 3.5043) was significantly more frequent in AB^+^VAC^−^ animals. Overall, the odds ratio for detecting *Salmonella* in any substrate or organ was significantly higher in the AB^+^VAC^−^ group animals (*p* = 0.0004, odds ratio: 5.9091). Treatment with tylosin can significantly increase the spread of a *Salmonella* infection, which is not observed after early *Lawsonia intracellularis* vaccination.

## 1. Introduction

Infections with *Lawsonia intracellularis* (*L. intracellularis*) leading to the so-called porcine proliferative enteropathy in pigs are one of the major reasons for economic losses in swine production, both in the case of clinical manifestations and in subclinical infections [1,2,3,4]. The chronic form of this enteropathy is often accompanied by moderate diarrhoea, to a lesser extent by anorexia, and by various degrees of inflammation of the terminal portion of the ileum [5,6,7]. The subclinical form as a somewhat milder version of the disease also worsens performance (lower feed intake, lower average daily weight gains, and a worse feed efficiency) [7,8,9,10,11]. The overall prolonged rearing and fattening period is a typical finding of these subclinical infections [1,12,13,14]. The acute form of the proliferative enteropathy causes black-red tarry faeces, anaemia, and sudden death in finishers, young adults or breeder pigs [7]. As a result, a high morbidity and high mortality in the affected groups of animals can occur [7]. All three forms of the disease can be a reason for the therapeutic and metaphylactic treatment with antibiotics or preventive vaccination [5,6,7].

Applying antibiotics in low doses as growth promoters has to be distinguished from therapeutic or metaphylactic treatments. Growth promoters, such as tylosin, are effective in the gram-positive spectrum and are supposed to lead to a reduction in the gastrointestinal commensal flora or to a shift towards a more gram-negative spectrum [15,16]. Interactions between the microbiome and susceptibility to *Salmonella* infections have been discussed [17]. Especially in poultry production, since the 1950s antimicrobials have been added to poultry feed at sub-therapeutical levels to minimise illness and promote growth [18]. Furthermore, there are fierce debates worldwide on whether or not this practice bears the consequences in terms of human health [18]. In this context, for example, an increased prevalence of *Salmonella* was reported, when experimentally challenged birds were fed diets containing low levels of antimicrobials [18]. However, there are also studies on turkeys, for example, demonstrating that *Salmonella* populations were significantly decreased when rations containing flavomycin, virginiamycin, or monensin were fed [18]. Additionally, in pigs, there are discussions that those animals receiving tylosin pose a public health concern due to a higher carriage of *Salmonella* [19]. There are European epidemiological studies, which see a clear risk of increased *Salmonella* seroprevalence when using antimicrobial growth promoters [20] or antibiotic substances for disease therapy [21]. In the first mentioned study from the Netherlands, the use of tylosin as an antimicrobial growth promoter in the finishing feed was associated with a higher *Salmonella* seroprevalence [20]. In the second study, a German epidemiological survey with a statistical analysis of results obtained from blood sample testing showed that administering antibiotics for therapeutic purposes increased the odds ratio by a factor of 5.21 (*p* < 0.001) compared to untreated pigs [21]. In addition, intensive contact with the pathogen itself could play a role [22]. The risk of *Salmonella* shedding at the end of the fattening period was increased when *L. intracellularis* seroconversion was seen during the fattening period [22]. In a study on 105 French swine herds, a statistically significant association was found (odds ratio 3.2, 90% confidence interval 1.4 to 7.2) between infections with *L. intracellularis* and the carriage of *Salmonella enterica* [22]. Therefore, it seems possible that *L. intracellularis* interacts with *Salmonella enterica* and/or other members of the gut microbiome and that these interactions lead to increased colonisation and the shedding of *Salmonella enterica* as hypothesised by some authors [17]. These authors assume that *L. intracellularis*, the cause of porcine proliferative enteropathy which colonises the ileum, and *Salmonella enterica* which colonises the colon and caecum, are both likely to have indirect interactions that might be mediated by other members of the gastrointestinal microbiome [17].

The public health aspects are of special importance as most human cases of salmonellosis (∼90%) are attributable to layers/eggs and pigs [23]. Salmonellosis is still a major cause of human bacterial gastroenteritis and the second most reported zoonosis in the European Union (EU) after campylobacteriosis [24]. In 2015, a total of 94,625 confirmed salmonellosis cases were reported by 28 EU member states, resulting in an EU notification rate of 21.2 cases per 100,000 population [24]. 

To date, there is no experimental study comparing the effects of the two possible treatments (antibiotic metaphylaxis or preventive vaccination) with regard to the spread of an experimental *Salmonella* infection in natural *L. intracellularis* infected pigs from herds with early infections. In our model with naturally infected piglets, the experimental *Salmonella* infection most closely reflects the conditions in practice under maximally standardised conditions. The aim of the investigations was to determine whether differences exist in the spread of an artificial *Salmonella* infection in groups with potentially subclinically *L. intracellularis* infected piglets, comparing animals that were either previously immunised as suckling piglets against the pathogen or therapeutically treated with an antibiotic against *L. intracellularis* just before the experimental infection with *Salmonella* Derby.

## 2. Materials and Methods

Animal experiments were performed in accordance with the German rules and regulations and approved by the Ethics Committee of Lower Saxony for the Care and Use of Laboratory Animals (LAVES: Niedersächsisches Landesamt für Verbraucherschutz und Lebensmittelsicherheit; reference: 33-9-42502-04-12/0902).

### 2.1. Origin of Animals and their Preparation and Selection on the Farm

The crossbred piglets for the trial came from one German farm with 420 sows of Danish genetics (damline: Danish Landrace 50% × Yorkshire 50% (DK); sire line: Pietrain, artificial insemination). The production system on the farm was organised at two-weekly intervals with 21 days of suckling in general with 450 to 500 piglets weaned per group. The piglets in the herd were regularly vaccinated (porcine circovirus 2, *Mycoplasma hyopneumoniae* and *Haemophilus parasuis*). In the context of regular and long-term screening programmes, the herd showed no signs of *Salmonella* and *Brachyspira* infections. The selection of the farm from which the animals came was made in a targeted manner. Three essential characteristics had to be fulfilled:

- In the past, the pathogen *L. intracellularis* had been repeatedly detected and was suspected of hindering the optimal performance on the farm.
- The farmer had to be in the process of testing a commercial vaccine in part of the herd on his own initiative in order to further improve animal performance.
- The trial phase of the partial vaccination of the herd had to be supervised by the herd supervising veterinary practice.

The farm from which the animals were collected fulfilled these criteria. Clinical symptoms of an *L. intracellularis* infection (confirmed by pathogen detection in faeces) were a common finding in piglets between the ages of seven to nine weeks. At the time of the investigations, the farmer was faced with the decision to establish a vaccination against *L. intracellularis* in the herd. The animals had not been vaccinated so far. Therefore, individual litters were vaccinated over several months in order for the farmer and veterinary surgeon to clinically observe the effects on the performance in piglet rearing and fattening.

During the trial period, every two weeks, approximately 50 piglets from a total of four to five complete litters were vaccinated with a commercial *L. intracellularis* live vaccine (Enterisol^®^Ileitis, Boehringer Ingelheim Vetmedica GmbH, Ingelheim/Rhine, Germany) on the 21st day of life. The vaccination was administered via oral drenching. Subsequently, the piglets were marked individually.

**Figure 1:**
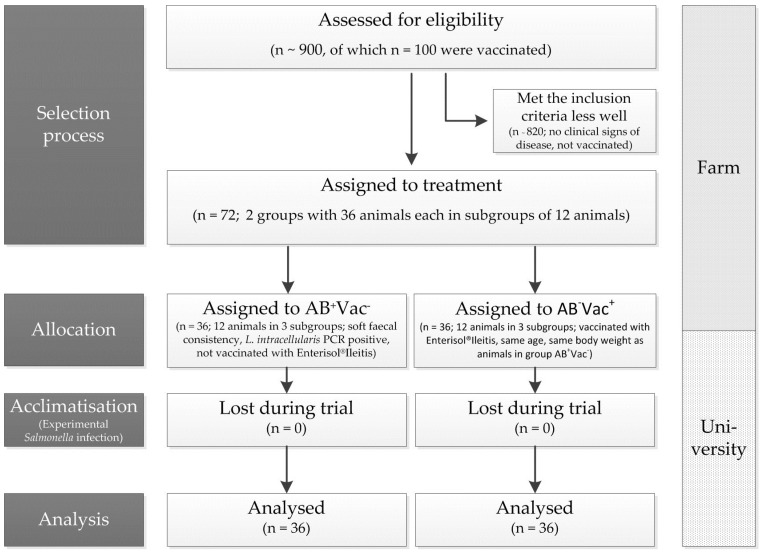
The clinical trial flow diagram on the selection process of animals on the farm for the experimental trial at the university with artificial *Salmonella* infection, allocation to groups (AB^+^VAC^−^: animals were treated with tylosin (10 mg/kg body weight) for seven days (AB^+^VAC^−^) during the trial; AB^−^VAC^+^: animals were vaccinated as suckling pigs by drenching (Enterisol^®^Ileitis)), as well as the number of animals.

After weaning, all 450 to 500 piglets in a group were housed in a piglet rearing compartment in boxes with a maximum of 30 animals on fully slatted floors. Non-vaccinated and vaccinated animals were mixed. From weaning to the presumed initial *L. intracellularis* infection time point, samples from the individual boxes were tested several times for their *L. intracellularis* status by real-time PCR using established methods [25]. A single-animal examination procedure started with positive proof (Figure 1). All piglets in a weaning group were clinically examined. Individual samples were collected from non-vaccinated conspicuous animals with regard to faecal quality (healthy pigs with moderate to soft faecal consistency, no diarrhoea) and analysed by means of real-time PCR depending on *L. intracellularis* status [25]. After obtaining the results, conspicuous animals were taken from the appropriate weaning group (subsequently called the “AB^+^VAC^−^ group” due to the antibiotic treatment necessary in the further course of the study). At the same time, vaccinated animals of the same age and identical weight were chosen from different boxes (subsequently called the AB^−^VAC^+^ group). In the course of the routine herd diagnostics, blood samples for *L. intracellularis* proof were simultaneously available from these animals (about four-five weeks after weaning). All the selected animals were culturally tested for *Salmonella* by rectal swabs to exclude the risk of being positive for *Salmonella* before starting the trials at the university. After obtaining the results, animals were transported to the Institute for Animal Nutrition, University of Veterinary Medicine Hannover, Foundation, Hannover, Germany.

### 2.2. Experimental Conditions, Pretreatment, and Infection Model

**Figure 2:**
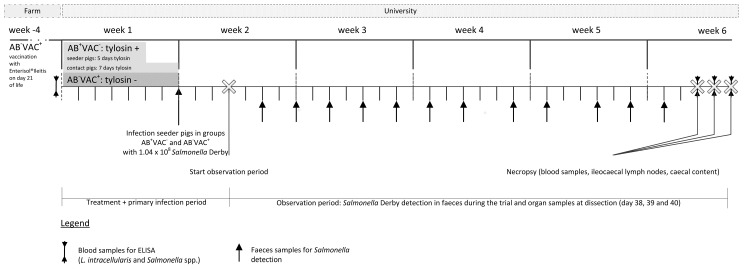
The infection model for the experimental *Salmonella* Derby infection in seeder pigs (experimentally infected) and contact pigs (secondarily infected); AB^+^VAC^−^ seeder − tylosin = two animals per group were administered tylosin (Tylan^®^ G 25%, Elanco Deutschland GmbH, Bad Homburg, active ingredient: tylosin phosphate, dosage: 10 mg/kg body weight orally) for five days; AB^+^VAC^−^ contact pigs−tylosin = ten pigs per group were administered tylosin (Tylan^®^ G 25%, Elanco Deutschland GmbH, Bad Homburg, active ingredient: tylosin phosphate, dosage: 10 mg/kg body weight orally) for seven days; seeder pigs in each group were returned to the respective group at day 10; after that, the observation period started. Pigs in the AB^−^VAC^+^ group had been vaccinated as suckling pigs by drenching (Enterisol^®^Ileitis) without further antibiotic treatment.

In total, 72 piglets (DK^x^Pietrain) were divided into two groups with three subgroups of 12 animals each. The first group was treated metaphylactically with tylosin (Tylan^®^ G 25%, Elanco Deutschland GmbH, Bad Homburg, Germany; dosage: 10 mg/kg body weight orally) for a minimum of five days (AB^+^VAC^−^; Figure 2), whereas the second group (vaccinated as suckling pigs by drenching (Enterisol^®^Ileitis)) was given no antibiotics (AB^−^VAC^+^). The drug was mixed into a small amount of feed each morning during the treatment period. This mixture was offered to the animals until complete ingestion before the rest of the daily feed was offered.

The boxes (3.00 × 2.15 m) were equipped with a nipple drinker, two stainless steel troughs (each 0.80 m long at one of the narrow sides of the box), and an infrared warming lamp. The boxes had a concrete floor. Additional boxes (3.00 × 1.05 m) were placed adjacent to the subgroup boxes. After five days, two pigs per group (later referred to as seeder animals) were housed in these separate boxes for the purpose of artificial *Salmonella* infection. The infection took place two days later, two days after ending the tylosin treatment of these seeder animals. The remaining animals of the AB^+^VAC^−^ group were administered tylosin for a further two days, i.e., a total of seven days. Therefore, it was guaranteed that at the time of artificial infection (seeder pigs), or rather, natural infection (contact pigs) the animals had been at least 48 without antibiotics.

The experimental infection was carried out in both groups in accordance with the model of Papenbrock et al. [26]. In each case, two seeder animals from one box of the subgroups from the AB^+^VAC^−^ and AB^−^VAC^+^ groups were infected. In total, three batches of infection broth were used (batch 1: 8.70 × 10^7^; batch 2: 1.10 × 10^8^; batch 3: 1.15 × 10^8^ per animal, respectively). The strain A147/85 of *Salmonella enterica* ssp. *enterica* serovar Derby (antigenic formula O 1, 4, [5], 12, H f, g [1,2]) used for infecting the seeder pigs was provided by the Institute of Microbiology of the University of Veterinary Medicine, the Hannover Foundation, where it was stored in a lyophilised form. This strain had been previously used successfully for experimental infection of pigs [26].

In order to increase the gastric pH and thus prevent damage to the germs by the very acidic stomach environment of pigs [27], the seeder animals were each given 50 g of feed in the morning. After about 15 min, another 50 g of feed was mixed with 10 mL of freshly prepared infection broth and presented to the pigs for ingestion. The amount of feed was kept to a minimum to ensure a fast and complete intake. After complete ingestion of the feed containing the infection broth, the animals were given food *ad libitum* as usual. After successfully detecting the infection strain by means of rectal swabs, the experimentally infected animals were returned to their original subgroups (ten days after starting the trials, three days after experimental infection). In doing so, contact animals came into contact with the pathogen. From this point onwards, a four-week follow-up examination using rectal swabs (2, 4, 6, 8, 10, 12, 15, 17, 19, 22, 24, and 26 days after returning the seeder pigs to the group) was followed by a necropsy of all animals at days 38–40 after starting the trials. In each group, after confirming the successful infection of the seeder pigs, 144 swab samples (12 per animal; 864 swabs in the entire study in six subgroups) were taken over the entire four-week experimental period.

### 2.3. Necropsy

At the day of dissection, the pigs had ad libitum access to diets starting at 06:00. Dissection started two hours later. Prior to being dissected, the animals were anaesthetised with neuroleptanalgesia by means of a combination of ketamine (Ursotamin^®^ 10%, Serumwerke Bernburg, Bernburg, Germany; active ingredient: ketamine hydrochloride, dosage: 15 mg/kg body weight intramuscularly) and azaperone (Stresnil^®^ 4% Janssen Animal Health, Neuss, Germany; active ingredient: azaperone, dosage: 2 mg/kg body weight intramuscularly). At dissection, a blood sample was taken intracardially from the pigs. After that, the pigs were euthanised with T61^®^ intracardially (Intervet, Unterschleißheim, Germany; active ingredients: tetracaine hydrochloride, embutramide, mebenzonium iodide, dosage: 0.6 mL/10 kg body weight).

### 2.4. Feed and Feed Analysis

The animals were given a complete diet (meal) for pigs of a common composition during the experiment (For Farmers-Bela GmbH, Langförden, Germany; Table 1). The diet contained wheat, barley, soybean meal, soybean oil, fried bread, wheat bran, calcium carbonate, monocalcium phosphate, sodium chloride, and minerals in descending order. No organic acids were added to the diet. The pigs were fed ad libitum once a day at 08:00.

The diets were analysed by standard procedures in accordance with the official methods of the VDLUFA [28]. The analyses were always performed in duplicate. The dry matter content was determined by drying to a constant weight at 103 °C. The crude ash was analysed by means of incineration in the muffle furnace at 600 °C for six hours. The total nitrogen content was determined by means of the analyser Vario Max (Elementar, Hanau, Germany), which operates according to the principle of a catalytic tube combustion (DUMAS combustion method). The molecular nitrogen formed by the reduction from nitric oxide was detected by a thermal conductivity detector and the nitrogen content was calculated by the device software. The crude protein content of the sample was calculated by multiplying it with a constant factor of 6.25. The crude fat content was determined after acid hydrolysis in the Soxhlet apparatus. The content of crude fibre was determined after washing in diluted acids and alkalis using established methods. The starch content was determined polarimetrically (Polatronic E, Schmidt und Haensch GmbH and Co., Berlin, Germany). The sugar content was analysed in accordance with Luff-Schoorl by titration with sodium thiosulphate. The mineral content was determined in accordance with the official methods [28] by atomic absorption spectrometry (UnicamSolaar 116, Thermo, Dreieich, Germany).

### 2.5. Lawsonia intracellularis and Salmonella Diagnostics

The serological tests (at the start of the trial and at necropsy) concerning *L. intracellularis* were carried out using a sandwich blocking ELISA in accordance with Keller et al. [29]. This ELISA has a specificity of 98.7% and a sensitivity of 96.5% and works with specific monoclonal antibodies. Cut-off values for the blocking ELISA test are given as per cent inhibition (PI) with a cut-off value of PI 30. Direct confirmation of the presence of *L. intracellularis* in the faeces of pigs in the AB^+^VAC^−^ group was made from the faeces collected on the farm via real-time PCR with established methods [25].

The serological tests concerning *Salmonella* were performed with an IDEXX Swine *Salmonella* Ab Test at the start of the trial (verification of being *Salmonella* unsuspicious) and at necropsy (an indicator of contact intensity with the pathogen). This test detects antibodies against the most common serotypes (B, C1, D) isolated in Europe, Asia, and America. The results are available as OD% values using IDEXX xChek^®^ software. The samples were considered positive if the optical density (OD) was ≥10%. 

For bacteriological *Salmonella* testing, the qualitative examination of different samples was carried out after liquid pre-enrichment (feed samples, organ samples). Feed and organ samples were non-selectively pre-enriched in peptone water (Oxoid, Wesel, Germany) for 24 h at 37 °C. Subsequently, the liquid selective Rappaport-Vassiliadis (RV) enrichment medium (Oxoid, Wesel, Germany) and Tetrathionate Brilliant Green Galle Broth (TBG; Merck, Darmstadt, Germany) were supplemented with 0.1 mL (for 9.9 mL RV) and 1 mL (for 9 mL TBG) of the suspension. These were further incubated for 48 h at 42 °C. Both after 24 h and after 48 h, one loop from each of the media was streaked onto both of the two selective nutrient media (Brilliance^TM^Salmonella, Oxoid, Wesel, Germany; brilliant green phenol red lactose sucrose agar—BPLS, Oxoid, Wesel, Germany). The culture medium was incubated at 37 °C (Brilliance) or 42 °C (BPLS). *Salmonella*-suspected colonies were determined by morphology, the staining of colonies, and the medium. *Salmonella*-suspected colonies were isolated and the surface antigens were characterised by means of rapid slide agglutination (anti-*Salmonella* O 4, anti-*Salmonella* O 5, Sifin GmbH, Berlin, Germany). Further differentiations were made using a Kligler agar (lactose negative, glucose positive, H_2_S-formation; [30]). For more far-reaching differentiation, colony material from the Kligler agar was subjected to further slide rapid agglutination for ascertaining the presence of flagellum antigens Hg and Hf (anti-*Salmonella* Hf, anti-*Salmonella* Hg, Sifin GmbH, Berlin, Germany). Both flagellum antigens are present in *S.* Derby.

### 2.6. Statistical Analyses

Analyses were carried out with the statistical software SAS, version 9.3 (SAS Institute, Cary, NC, USA), using SAS^®^ Enterprise Guide 5.1.

The comparison for quantitative variables between groups (independent samples; AB^+^VAC^−^, AB^−^VAC^+^) and within a group (paired *t*-test) was carried out by two-sample *t*-tests (BW; ADWG; FCR (normal distribution was assumed for *n* = 3)) for normal distributed data and by means of the Wilcoxon test in non-normally distributed data.

The analysis of the qualitative features (*Salmonella* detection in rectal swabs, caecal content, ileocaecal lymph nodes) was carried out using the Pearson’s chi-square homogeneity test. The odds ratio was also calculated for group comparison.

Differences were taken to be statistically significant when *p* < 0.05.

## 3. Results

The investigations ran without any abnormalities dependent on animal health. Even after the experimental infection with *S.* Derby, there were no clinical signs of infection. There were no animal losses during the trial.

### 3.1. Performance

The body weights at the start of the investigations, the average daily feed intake, the final body weight, the average daily weight gain, and the feed conversion ratio did not differ between groups (Table 2).

### 3.2. Serological and Microbiological Salmonella Statuses

The non-vaccinated animals (AB^+^VAC^−^) showed significantly higher *L. intracellularis* antibody titres at the time of selection of the animals on the farm compared to the vaccinated group and were characterised as serologically positive (Ø PI values at group level ≥30; Table 3). The vaccinated animals, however, were serologically negative. After transport to the university, at the start of the trials, the animals were regrouped in new boxes. At the end of the experiment, the mean *L. intracellularis,* PI-values were significantly higher in both groups, whereas the final values in the AB^−^VAC^+^ group were significantly higher than in the AB^+^VAC^−^ group.

The serological *Salmonella* status between the groups did not differ at the beginning of the investigations. Both groups scored average within the range that is considered negative by the test kit manufacturer (OD% values < 10). At the end of the experiment, the mean serological values in the AB^+^VAC^−^ group were significantly higher (AB^+^VAC^−^—OD%: 25.3 ± 26.3) than in the AB^−^VAC^+^ group (OD%: 14.1 ± 16.3). At the end of the experiment, 19 out of 33 pigs in the AB^+^VAC^−^ group and 15 out of 33 pigs in the AB^−^VAC^+^ group showed an OD% value higher than or equal to ten. Three samples in each group could not be examined.

All primary infected animals showed a *Salmonella* excretion in the faeces before being returned to the group. Additionally, during the trial, these animals again showed *Salmonella* shedding. *Salmonella* Derby was significantly more frequently detected in faecal samples from the AB^+^VAC^−^ group throughout the experiment (Table 4). The same applied to the number of positive animals regarding the faeces and the *Salmonella* prevalence in samples of ileocaecal lymph nodes and caecal content.

**Figure 3:**
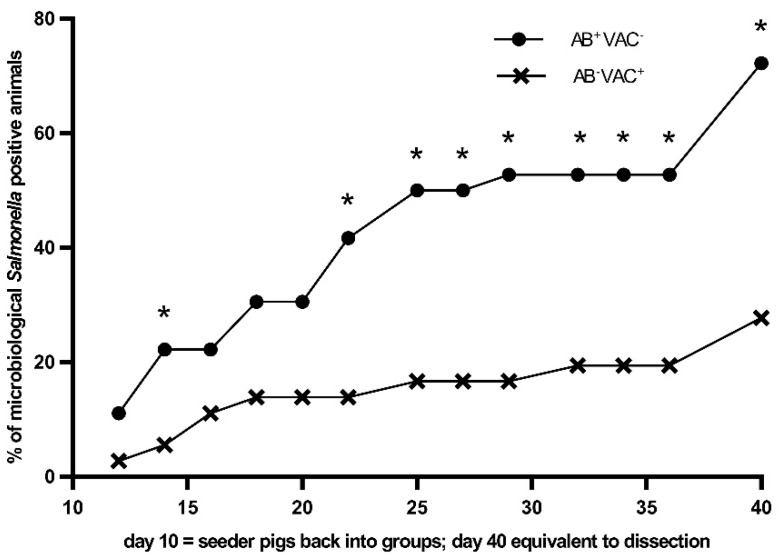
The proportion of positive samples depending on the microbiological *Salmonella* Derby detection during the observation period; percentage of positive samples after the seeder pigs had been returned to the contact groups (day 10 = seeder pigs back in groups) in tylosin-treated (AB^+^VAC^−^) and vaccinated animals (AB^−^VAC^+^) up to necropsy (finished at day 40).

If one of the microbiologically tested samples was *Salmonella* positive, the animal was classified as *Salmonella* positive overall. Therefore, the number of pigs (both seeder and contact pigs) which were detected positive in a minimum of one sample after being returned to the subgroup was significantly higher in the AB^+^VAC^−^ group at days 4, 12, 15, 17, 19, 22, 24, 26, and at necropsy (Figure 3).

### 3.3. The Interaction between Serological and Microbiological Salmonella Status

The serological *Salmonella* status of animals prior to the start of the study was identical for animals which remained negative or became positive after the experimental *Salmonella* infection during the study (Table 5).

The comparison of animals that were considered *Salmonella* negative with respect to the defined samples (faeces, ileocaecal lymph node, caecal content, sum of all locations) showed significantly lower serological *Salmonella* responses in the ELISA than animals in the AB^−^VAC^+^ group at the end of the experiment for all sample types (Table 6). In addition, a significant difference in the serological response to *Salmonella* was seen in this group when comparing animals that were either negative (OD%: 11.2 ± 13.1) or positive (OD%: 42.7 ± 5.03) for the detection of *Salmonella* in the caecum.

The comparison of animals depending on Δ-values concerning serological *Salmonella* responses in the ELISA showed significantly lower results for animals with a *Salmonella* negative status in ileocaecal lymph nodes and in the caecal content in the AB^−^VAC^+^ group (Table 7). In the AB^−^VAC^+^ group, the Δ-values concerning serological *Salmonella* responses were also significantly lower in animals with a negative *Salmonella* status in the caecal content in comparison to pigs where *Salmonella* was found in the caecal content.

## 4. Discussion

*Lawsonia intracellularis* is of great importance as a causative agent of digestive diseases in today’s pig production [1,2,3,4,31]. Nowadays, vaccination or antibiotic treatment are the two alternatives used to deal with the pathogen. The antibiotic treatment of *L. intracellularis* infections is discussed against the background regarding whether the treatment can accelerate the spread of zoonotic-relevant pathogens like *Salmonella*. To deal with these issues, artificial *Salmonella* infection experiments were conducted with naturally *L. intracellularis*-infected piglets.

### 4.1. Effects on Performance

In the present study, no significant differences concerning performance data could be seen. The animals were the same age and weight at the start of the experiment. The one-week treatment with tylosin did not have a positive or a negative effect on ADWG and FCR. For tylosin, the growth-promoting effects are known, especially from earlier times when tylosin was still used as a growth promotor [32,33]. In a study on pigs at 38 to 58 days of age reduction, the feed intake was 3.79% after experimental infection (1.26 × 10^10^ *L. intracellularis* organisms in the inoculum) in comparison with animals treated with 50 ppm tylvalos for 14 further days after experimental infection [34]. In terms of performance, in our study, there was no significant difference between the experimental groups (AB^+^VAC^−^: therapeutically 10 mg/kg BW, AB^−^VAC^+^: prophylactic vaccination). Therefore, in the present study, it can be assumed that both alternatives work equally well in terms of performance.

### 4.2. Lawsonia intracellularis and Salmonella Infection

The pigs used in these trials came from a farm with a history of early *L. intracellularis* infections. Animals in the AB^+^VAC^−^ group had already shown a serological response immediately before the start of the experiments with artificial *Salmonella* infections, which indicated an intensive field contact. The PCR samples of faecal material in the AB^+^VAC^−^ group support this finding. By contrast, animals in the AB^−^VAC^+^ group were serologically negative at the start. With regrouping after transport, a significant increase in antibody levels was seen. At the end of the experiments, the animals vaccinated as suckling piglets against *L. intracellularis* (AB^−^VAC^+^) showed the highest antibody titers. Intensive contact with the pathogen fosters the antibody response in pigs. Positive correlations (Pearson’s correlation coefficient) were observed between antibody concentrations in pigs at 21 days post-infection (orally dosed at 6 weeks of age) and the number of *L. intracellularis* shed 14 days (*r* = 0.52), 17 days (*r* = 0.58), and 21 days (*r* = 0.50) after infection [11]. Additionally, serum titers were higher in pigs challenged with a pathogenic isolate than in those exposed to the vaccine, which ranged from 30 to 3820 and from 30 to 480, respectively [35]. In most cases, pigs become seropositive two-three weeks after primary inoculation with *L. intracellularis* [36]. In vaccinated pigs, sometimes there can be a delayed onset of seroconversion [35]. In the named study, the authors explained the delayed seroconversion and the lower IgG titers observed in the pigs in the vaccine group compared to the animals in the pathogenic isolate group as being caused by a combination of factors, such as a lower concentration of *L. intracellularis* organisms in the vaccine compared to the pathogenic inoculum (5.3 × 10^5^ and 1.76 × 10^8^ organisms/mL, respectively), the route of administration (drinking water for the vaccine group and intragastrically for the pathogenic isolate group), and the immunogenicity of the isolate [35]. The higher antibody titers in the vaccinated group in this study may be explained by the fact that systemic antibody responses were boosted following challenge [37]. This development of the serological measurements clearly shows that the model worked well. In both groups, we had a serological reaction within the experimental period, indicating a more intensive contact with *L. intracellularis*.

Regarding the serological *Salmonella* status, all animals were serologically negative at the beginning of the experiments. During the trial, serological reactions to *Salmonella* increased significantly in both groups. Nevertheless, individual animals showed titers above the cut-off value already at the start of the experiment. In piglet rearing, maternal antibodies to *Salmonella* can be detected up until the seventh week of life [38]. Individual positive ELISA responses in both experimental groups at the start of the experiment are therefore likely to be due to the presence of maternal antibodies.

At the end of the trial, the prevalence of serologically positive samples was significantly higher in the AB^+^VAC^−^ group. A major advantage of serodiagnostics compared with bacteriological tests is those specific IgG antibodies generally persist, whereas *Salmonella* are excreted intermittently and pigs may harbour infections without excreting *Salmonella* over a prolonged period [39]. However, it should be mentioned that these tests are not suitable as an individual pig test because not all pigs seroconvert [39]. 

The odds ratio for positive *Salmonella* detection in individual samples or animals in general (independent of location, i.e., in faeces, ileocaecal lymph nodes, caecal content), was greatly increased in the AB^+^VAC^−^ group. This observation is in line with the results from European epidemiological studies, which see a clear risk of increased *Salmonella* seroprevalence when using antibiotic substances, both in the lower dosage as growth promotor or in therapeutic dosages [20,21] In the German study, the application of antibiotics increased the odds ratio for being serologically *Salmonella* conspicuous by a factor of 5.21 (*p* < 0.001) compared to untreated pigs [21]. A direct effect of tylosin on the commensal gastrointestinal flora has already been described for concentrations in the range of a body weight of 2 mg/kg [15]. In swine, the adverse effect of antibiotic treatment on gut flora persisted for only about a week, but animals are five to six times more susceptible to gastrointestinal tract infections during this time [40]. According to a US study, using the antimicrobial growth promoter tylosin did not pose a public health risk. There was no effect on the carriage of *S. enterica* [19]. From the point of view of the present investigations, it can be assumed that the antibiotic treatment fostered the spread of the *Salmonella* infection. However, it cannot be ruled out that the vaccination itself has a protective effect. Vaccination against *L. intracellularis* at three weeks of age significantly reduced *S.* Typhimurium shedding (*p* < 0.05) in co-infected animals in comparison to the co-infected group without vaccination and the group challenged with *S.* Typhimurium alone [41]. Twenty-one days post vaccination, the animals were challenged with a pure culture of *L. intracellularis* (2 × 10^9^ organisms per pig; strain PHE/MN1-00). One week post-*L. intracellularis* challenge, the pigs were challenged orally with *S.* Typhimurium (strain 798; 1 × 10^8^ organisms per pig) [41]. At seven days post-infection, the co-challenged non-vaccinated group shed 2.94 log_10_ *S.* Typhimurium organisms per gramme faeces, while the vaccinated co-challenged group shed 0.82 log_10_ *S.* Typhimurium organisms per gramme faeces (*p* = 0.003) [41]. These results indicate that the vaccination against *L. intracellularis* impacts the microbiome and reduces shedding of *S.* Typhimurium in co-infected animals [41]. These previous findings are in agreement with the results of the present study. In our study, the animals were also vaccinated against *L. intracellularis* in the suckling pig phase, selected about three weeks later and transported from the farm to the university. On arrival, the animals were regrouped and thus the *L. intracellularis* infection was re-established. It is known that regrouping and/or mixing animals from different herds in the finisher unit might increase the rate of transmission of *L. intracellularis* [42]. In both groups in our trial, the serological response to *L. intracellularis* increased significantly during the trial. In week two at the institute, the experimental infection with *S.* Derby took place. This experimental model is therefore comparable in principle to the design described above [41]. Therefore, this study supports the recommendation that the *L. intracellularis* vaccination may be used as a novel tool to aid the control of *Salmonella* on swine farms, as well as using it as an alternative measure to reduce the need for the antibiotic treatment of pigs, thereby improving food safety [41].

### 4.3. Interactions between Serological and Microbiological Findings

At the end of the experiment, animals with a negative status depending on different samples (faeces, ileocaecal lymph nodes, caecal content, one of the named samples positive for *S.* Derby) from the AB^−^VAC^+^ group had significantly lower *Salmonella* antibody values in the blood in each case than animals from the AB^+^VAC^−^ group. The delta values between the end of the experiment and the start of the experiment were also significantly lower for animals with negative findings in the lymph nodes and the caecal content in the AB^−^VAC^+^ group compared to the AB^+^VAC^−^ group.

High antibody levels also reflect a certain degree the history of cultural detection of *Salmonella*. According to the results of a previous experimental *Salmonella* infection study (4.4 × 10^9^ CFU *Salmonella* Typhimurium DT104), the majority of pigs produced no anti-*Salmonella* immunoglobulin G within the third week in spite of high *Salmonella* excretion rates [39]. Afterwards, all pigs showed a high seroprevalence, but in contrast, a low prevalence of *Salmonella* Typhimurium DT104 was shown in the faeces [39]. Another study on 1658 finishers from 167 herds suspected that there was a correlation between the herd seroprevalence (cut-off 20 OD%) and the probability of *Salmonella* isolation from 25 g of caecal content [43]. Overall, pigs with *Salmonella-*positive caecal content showed the highest mean optical densities in meat juice in another study, namely, 37.1 OD% (*n* = 124; [44]). Completely microbiologically negative animals (*n* = 178) instead had an LsMean OD% of 10.9 [44]. From these results, one can conclude that the differences in bacteriological findings in the present study are not overestimated because microbiologically negative animals from the AB^+^VAC^−^ group also had significantly higher *Salmonella* antibody values.

## 5. Conclusions

In conclusion, the present results clearly show that the antibiotic therapy of an infection with *L. intracellularis* leads to a higher susceptibility to the *S.* Derby infection in comparison to the alternative prophylactic vaccination. In the AB^+^VAC^−^ group pretreated with antibiotics, *Salmonella* spreading significantly increased after experimental infection compared to the AB^−^VAC^+^ group. In vaccinated pigs, therapy is not required. Therefore, with regard to the prevalence of *Salmonella* infections in pigs, excellent protection against *Salmonella* is expected from the vaccine.

## Figures and Tables

**Table 1:** The analysed nutrient content and energy of the diet for rearing the piglets.

Chemical Composition		Content *
Crude ash	(g/kg DM)	54.5 ± 1.15
Crude fat		34.9 ± 2.40
Crude fibre		37.9 ± 5.32
Crude protein		206 ± 3.61
Starch		466 ± 2.31
Ca		9.65 ± 0.54
P		6.27 ± 0.06
Cu	(mg/kg DM)	27.2 ± 3.61
Zn		219 ± 27.5
Metabolisable Energy	(MJ/per kg diet)	13.5 ± 0.25

* averages of the three charges.

**Table 2:** The performance parameters in tylosin-treated (AB^+^VAC^−^) and vaccinated animals (AB^−^VAC^+^).

Item		AB^+^VAC^−^ *	AB^−^VAC^+^ *
BW start	(kg)	23.1 ± 1.85	23.2 ± 2.38
BW necropsy		56.5 ± 4.05	56.2 ± 4.80
ADFI		1.84 ± 0.13	1.80 ± 0.07
ADWG	(g)	798 ± 72.1	789 ± 88.5
FCR	(kg diet/kg gain)	2.31 ± 0.15	2.28 ± 0.09

AB^+^VAC^−^ = tylosin treated before artificial *S.* Derby infection, non-vaccinated; AB^−^VAC^+^ = vaccinated with Enterisol^®^Ileitis in the suckling period (day 21); BW = Body Weight; ADFI = Average Daily Feed Intake; ADWG = Average Daily Weight Gain; FCR = Feed Conversion Ratio; * no statistical differences between parameters.

**Table 3:** The mean antibody titres in the blood (*L. intracellularis* and *Salmonella* Derby) of tylosin-treated (AB^+^VAC^−^) and vaccinated animals (AB^−^VAC^+^).

Item	Time Point/Period	AB^+^VAC^−^	AB^−^VAC^+^
PI values *L. intracellularis* blocking ELISA	start	31.5 ^aB^ ± 27.0	9.00 ^bB^ ± 18.9
	necropsy	46.3 ^bA^ ± 17.3	59.9 ^aA^ ± 20.1
	Δ	11.0 ± 35.2	45.9 ^a^ ± 35.9
*Salmonella* OD%	start	7.14 ^B^ ± 11.8	3.58 ^B^ ± 5.85
	necropsy	25.3 ^aA^ ± 26.3	14.1 ^bA^ ± 16.3
	Δ	16.1 ± 27.9	9.33 ± 15.0

AB^+^VAC^−^ = treated with tylosin before artificial *S.* Derby infection, non-vaccinated; AB^−^VAC^+^ = vaccinated with Enterisol^®^Ileitis in the suckling period (day 21); PI = percent inhibition, cut-off values for the *L. intracellularis* blocking ELISA test with a cut-off value of PI 30; IDEXX Swine *Salmonella* Ab Test: OD% values were considered positive if the optical density (OD) was ≥10%; upper case letters (^A, B^) signify differences in columns (vertical) within a group for the analysed parameters at *p* < 0.05; lower case letter (^a, b^) signify differences in rows (horizontal) between groups depending on the parameter at *p* < 0.05.

**Table 4:** The microbiological detection of *Salmonella* in tylosin-treated (AB^+^VAC^−^) and vaccinated animals (AB^−^VAC^+^).

Item	Time Point/Period	n/Group	AB^+^VAC^−^ (n *S*.+)	AB^−^VAC^+^ (n *S*.+)	*p*-Value	OR	95% Confidence Limit
Faeces	start	432	36	10	<0.0001	3.8364	1.88–7.83
	necropsy	36	19	7	0.0032	4.6303	1.61–13.3
Ileocaecal lymphnode	Δ	36	13	5	0.0295	3.5043	1.09–11.2
Caecal content	start	36	6	3	0.2850	2.2000	0.51–9.58
∑ animals with pos. samples	necropsy	36	25	10	0.0004	5.9091	2.13–16.3

AB^+^VAC^−^ = treated with tylosin before artificial *S.* Derby infection, non-vaccinated; AB^−^VAC^+^ = vaccinated with Enterisol^®^Ileitis in the suckling period (day 21); n *S*.+ = number of *Salmonella-*positive samples at microbiological detection; OR = Odds Ratio.

**Table 5:** The serological *Salmonella* status (OD%) before the start of the experiments depending on later *Salmonella* detection in tylosin-treated (AB^+^VAC^−^) and vaccinated animals (AB^−^VAC^+^).

Sample	Microbiological S.-Status	Serological *S*.-Status at Start (OD%) *			
		AB^+^VAC^−^		AB^−^VAC^+^	
		n	Mean ± SD	n	Mean ± SD
Faeces	negative	17	8.82 ± 13.7	29	3.66 ± 5.91
	positive	19	5.63 ± 9.92	7	3.29 ± 6.05
Ileocaecal lymphnode	negative	23	5.96 ± 8.83	31	3.29 ± 5.65
	positive	13	9.23 ± 16.0	5	5.40 ± 7.37
Caecal content	negative	30	8.03 ± 12.7	33	3.67 ± 6.07
	positive	6	2.67 ± 2.25	3	2.67 ± 2.89
Sum of all locations	negative	11	6.18 ± 9.39	26	3.12 ± 5.48
	positive	25	7.56 ± 12.9	10	4.80 ± 6.88

AB^+^VAC^−^ = treated with tylosin before artificial *S.* Derby infection, non-vaccinated; AB^−^VAC^+^ = vaccinated with Enterisol^®^Ileitis in the suckling period (day 21); IDEXX Swine *Salmonella* Ab Test: OD% values were considered positive if the optical density (OD) was ≥10%; * no statistical differences between parameters.

**Table 6:** The serological *Salmonella* status (OD%) at necropsy in relation to *Salmonella* detection during the trial in tylosin-treated (AB^+^VAC^−^) and vaccinated animals (AB^−^VAC^+^).

Sample	Microbiological S.-Status	Serological S.-Status at Necropsy (OD%)			
		AB^+^VAC^−^		AB^−^VAC^+^	
		n	Mean ± SD	n	Mean ± SD
Faeces	negative	15	28.8 ^a^ ± 30.2	26	13.4 ^b^ ± 16.4
	positive	18	22.4 ± 23.1	7	16.6 ± 17.3
Ileocaecal lymphnode	negative	21	21.9 ^a^ ± 23.9	29	10.8 ^b^ ± 11.7
	positive	12	31.3 ± 30.3	4	37.8 ± 26.7
Caecal content	negative	27	23.7 ^a^ ± 26.5	30	11.2 ^bB^ ± 13.1
	positive	6	32.5 ± 26.6	3	42.7 ^A^ ± 5.03
Sum of all locations	negative	10	31.9 ^a^ ± 31.2	24	9.88 ^b^ ± 10.7
	positive	23	22.5 ± 24.1	9	25.3 ± 23.3

AB^+^VAC^−^ = treated with tylosin before artificial *S.* Derby infection, unvaccinated; AB^−^VAC^+^ = vaccinated with Enterisol^®^Ileitis in the suckling period (day 21); IDEXX Swine *Salmonella* Ab Test: OD% values were considered positive if the optical density (OD) was ≥10%; upper case letters (^A, B^) signify differences in columns (vertical) within a group for analysed parameters at *p* < 0.05; lower case letters (^a, b^) signify differences in rows (horizontal) between groups depending on parameter at *p* < 0.05.

**Table 7:** The delta-values in the serological *Salmonella* status (OD%) between the start of the experiment and necropsy in relation to *Salmonella* detection during the trial in tylosin-treated (AB^+^VAC^−^) and vaccinated animals (AB^−^VAC^+^).

Sample	Microbiological S.-Status	∆ serological S.-Status (OD%)			
		AB^+^VAC^−^		AB^−^VAC^+^	
		n	Mean ± SD	n	Mean ± SD
Faeces	negative	15	22.2 ± 30.4	26	9.46 ± 14.8
	positive	18	17.9 ± 20.3	7	13.3 ± 17.9
Ileocaecal lymphnode	negative	21	16.6^a^ ± 22.3	29	7.37^b^ ± 12.2
	positive	12	25.7 ± 29.5	4	31.3 ± 21.2
Caecal content	negative	27	17.7^a^ ± 24.8	30	7.30^bB^ ± 12.5
	positive	6	29.8 ± 25.6	3	40.0^A^ ± 2.65
Sum of all locations	negative	10	25.2 ± 32.0	24	6.58 ± 11.2
	positive	23	17.6 ± 21.9	9	20.1 ± 20.6

AB^+^VAC^−^ = treated with tylosin before artificial *S.* Derby infection, non-vaccinated; AB^−^VAC^+^ = vaccinated with Enterisol^®^Ileitis in the suckling period (day 21); IDEXX Swine *Salmonella* Ab Test: OD% values were considered positive if the optical density (OD) was ≥10%; upper case letters (^A, B^) signify differences in columns (vertical) within a group for analysed parameters at *p* < 0.05; lower case letters (^a, b^) signify differences in rows (horizontal) between groups depending on parameter at *p* < 0.05.
